# Lysine Deprivation Suppresses Adipogenesis in 3T3-L1 Cells: A Transcriptome Analysis

**DOI:** 10.3390/ijms24119402

**Published:** 2023-05-28

**Authors:** Leo Man-Yuen Lee, Zhi-Qiang Lin, Lu-Xi Zheng, Yi-Fan Tu, Yik-Hing So, Xiu-Hua Zheng, Tie-Jun Feng, Xi-Yue Wang, Wai-Ting Wong, Yun-Chung Leung

**Affiliations:** 1 Department of Applied Biology and Chemical Technology, The Hong Kong Polytechnic University, Hung Hom, Kowloon, Hong Kong, China; 2 Lo Ka Chung Research Centre for Natural Anti-Cancer Drug Development and State Key Laboratory of Chemical Biology and Drug Discovery, The Hong Kong Polytechnic University, Hung Hom, Kowloon, Hong Kong, China; 3 School of Biomedical Science, The Chinese University of Hong Kong, Shatin, New Territory, Hong Kong, China; 4 Department of Obstetrics and Gynecology, The Chinese University of Hong Kong, New Territory, Hong Kong, China; 5 Shenzhen Research Institute, The Hong Kong Polytechnic University, Shenzhen 518000, China

**Keywords:** lysine deprivation, amino acid depletion, adipogenesis inhibition, anti-obesity

## Abstract

Growing evidence proves that amino acid restriction can reverse obesity by reducing adipose tissue mass. Amino acids are not only the building blocks of proteins but also serve as signaling molecules in multiple biological pathways. The study of adipocytes’ response to amino acid level changes is crucial. It has been reported that a low concentration of lysine suppresses lipid accumulation and transcription of several adipogenic genes in 3T3-L1 preadipocytes. However, the detailed lysine-deprivation-induced cellular transcriptomic changes and the altered pathways have yet to be fully studied. Here, using 3T3-L1 cells, we performed RNA sequencing on undifferentiated and differentiated cells, and differentiated cells under a lysine-free environment, and the data were subjected to KEGG enrichment. We found that the differentiation process of 3T3-L1 cells to adipocytes required the large-scale upregulation of metabolic pathways, mainly on the mitochondrial TCA cycle, oxidative phosphorylation, and downregulation of the lysosomal pathway. Single amino acid lysine depletion suppressed differentiation dose dependently. It disrupted the metabolism of cellular amino acids, which could be partially reflected in the changes in amino acid levels in the culture medium. It inhibited the mitochondria respiratory chain and upregulated the lysosomal pathway, which are essential for adipocyte differentiation. We also noticed that cellular interleukin 6 (IL6) expression and medium IL6 level were dramatically increased, which was one of the targets for suppressing adipogenesis induced by lysine depletion. Moreover, we showed that the depletion of some essential amino acids such as methionine and cystine could induce similar phenomena. This suggests that individual amino acid deprivation may share some common pathways. This descriptive study dissects the pathways for adipogenesis and how the cellular transcriptome was altered under lysine depletion.

## 1. Introduction

Obesity is a severe public health challenge in the 21st century, and its prevalence is increasing at an alarming rate [1]. It has also been associated with many other metabolic disorders, including diabetes, cardiovascular diseases, and hypertension, leading to a poor quality of life and a severe social burden [2]. One key factor contributing to the fat mass expansion is excessive nutrients, especially fat and carbohydrates. However, increasing evidence has shown that dietary protein and levels of individual amino acids are also crucial for adipogenesis and energy expenditure [3,4,5]. There is growing interest in controlling body weight via regulating dietary micronutrient levels, especially essential amino acids (EAAs). There are nine EAAs: histidine, isoleucine, leucine, lysine, methionine, phenylalanine, threonine, tryptophan, and valine. Research showed that histidine, leucine, lysine, threonine, and tryptophan supplementation could reduce body weight gain and visceral fat deposition in diet-induced obese rats [6,7,8,9,10]. In contrast, a depletion of all nine EAAs individually in the diet, similarly, could reduce body fat mass. The depletion of some EAAs could even lower glucose and triglyceride levels, and improve insulin sensitivity in normal and obese mouse models [3,11,12]. These paradoxical effects of amino acid imbalance on obesity prevention are still not fully understood.

Adipogenesis occurs when the body is overloaded with nutrients, contributing to a positive energy balance. It is a multi-step process involving stem cells’ differentiation into mature, lipid-containing adipocytes [13]. There are two phases of adipogenesis, determination and terminal differentiation. The determination step is the process of mesenchymal stem cells committing to the preadipocytes, while terminal differentiation starts when preadipocytes differentiate into mature adipocytes. Peroxisome proliferator-activated receptor gamma (PPARg) and CCAAT-enhancer-binding protein alpha (C/EBPa) are known to be central to white adipose tissue adipogenesis [14]. Other signaling pathways in the regulation of adipogenesis have been identified, such as activation of insulin-like growth factor 1 (IGF1) [15,16], cyclic adenosine monophosphate (cAMP) [17], and bone morphogenetic proteins (BMPs) signaling [18]. In contrast, inhibitory effects were observed from transforming growth factor beta (TGFb) signaling [19], WNT signaling [20], and retinoblastoma protein (Rb) signaling [21]. The presence of branched-chain amino acids (BCAA), including leucine, isoleucine, and valine, has been proven essential for driving adipogenesis in 3T3-L1 cells as they serve as a source of acetyl-coenzyme A for lipogenesis [22,23]. The inhibition of BCAA catabolism obstructs adipogenesis. However, the effect of other essential amino acids on the adipogenic process has rarely been studied. L-lysine is an essential amino acid that plays a vital role in anxiogenic behavior in humans and rodents [11,24]. Interestingly, the effect of lysine depletion on adipogenesis is controversial, particularly species-specific. Low lysine treatment stimulates bovine stromal vascular cells and intramuscular preadipocyte adipogenesis [25,26]. In mice, low lysine concentrations suppress adipogenesis in 3T3-L1 preadipocytes via the inhibition of PPARg [27], and the underlying mechanisms of lysine-deprivation-induced transcriptome changes in adipocyte differentiation have not been solved.

**Figure 1:**
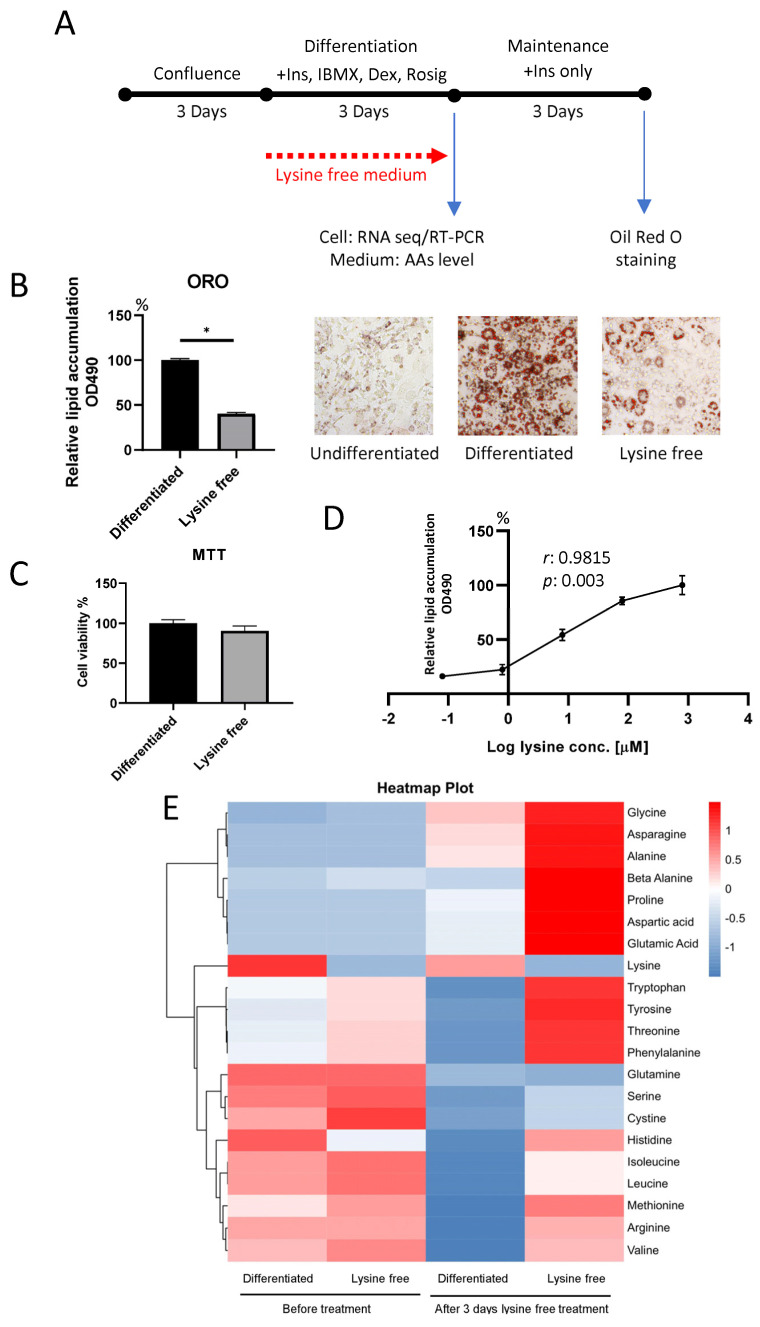
Lysine depletion during the 3T3-L1 cell differentiation period suppressed adipogenesis and altered amino acid metabolism. (**A**) The schematic diagram demonstrates the experimental design; lysine-free medium was applied only in the differentiation period. (**B**) Reduction in lipid formation in lysine-free treatment was found 3 days after differentiation by Oil-Red O (ORO) staining. (**C**) Cell viability of the cells determined by MTT assay. Data are expressed as means ± SE with n = 3 in each condition. * *p* <0.05 using the Mann–Whitney U test. (**D**) 3T3-L1 cell response to lysine concentration in a dose-dependent manner (800, 80, 8, 0.8, and 0.08 μM). Data are expressed as means ± SE with n = 3 in each condition. Correlation was analyzed using Pearson’s correlation test. (**E**) Heatmap of amino acid content in culture medium before and after treatment.

We herein performed RNA sequencing on 3T3-L1 preadipocytes in different statuses, including undifferentiated, differentiated, and differentiated with lysine depletion (Figure 1A). The transcriptome data were compared among groups with Kyoto Encyclopedia of Genes and Genomes (KEGG) database enrichment to screen out possible pathways induced by lysine deprivation and distinguish which pathways were critical for adipogenesis, and which pathways were triggered by the differentiation process but were irrelevant to the adipogenic process. We also discovered that the suppression of adipogenesis is not limited to lysine depletion but also applies to other essential amino acids such as methionine and cystine.

## 2. Results

### 2.1. Lysine Deprivation Suppressed Adipogenesis

**Figure 2:**
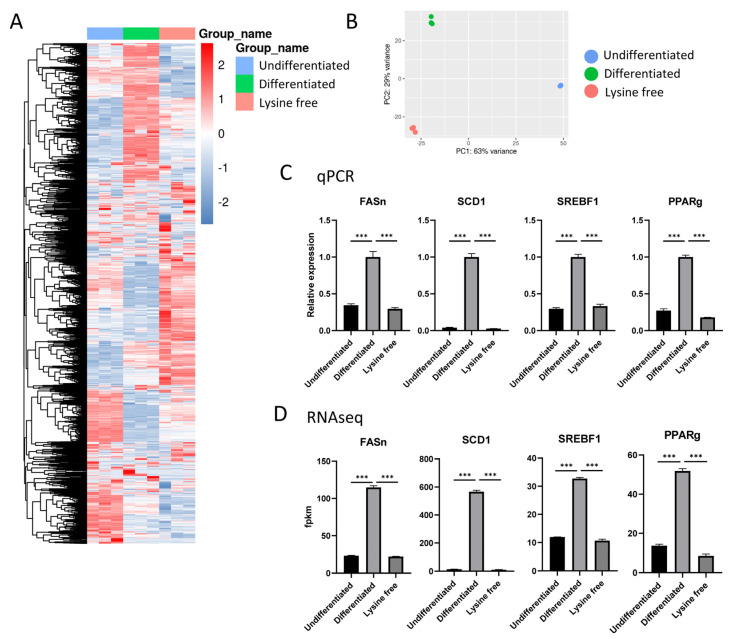
RNAseq data were highly consistent within the group and coherent with real-time qPCR data. (**A**) Heatmap of all genes in samples of the 3 groups, including undifferentiated, differentiated, and lysine-free (Lysfree). (**B**) PCA plot showing that the samples within the group were highly stable. (**C**) qPCR of the representative adipogenic genes FASn, SCD1, SREBF1, and PPARg. (**D**) The expression level of the adipogenic genes in fpkm from RNAseq analysis. Data are expressed as means ± SE with n = 3 in each condition. *** *p* < 0.001 using the one-way ANOVA followed by the Bonferroni test.

To examine the effect of lysine starvation on 3T3-L1 cell differentiation, cells were cultured in a differentiation medium containing dexamethasone (Dex), 3-isobutyl-1-methylxanthine (IBMX), rosiglitazone (Rosig), and insulin in the presence or absence of 800 μM lysine (Figure 1A). Oil-Red O (ORO) staining was performed 3 days after the differentiation, demonstrating that the adipogenesis process was significantly inhibited in cells treated with lysin-deficient medium (Figure 1B). Coherent with the suppressed differentiation, the expressions of adipogenic and lipogenic genes such as fatty acid synthase (FASn), stearoyl-coenzyme A desaturase (SCD1), sterol regulatory element binding transcription factor 1 (SREBF1), and PPARg were found to be dramatically downregulated (Figure 2C,D). Cell viability assay demonstrated that there was no difference between cells cultured in normal and lysine-free medium (Figure 1C), proving that the suppression of adipogenesis is not related to reduced cell number. We also confirmed that cells could respond to lysine in a dose-dependent manner (Figure 1D), suggesting that cells sense the level of lysine in the environment. To ensure the absence of lysine in the culture medium, we measured the levels of major amino acids in the medium before and after lysine-free treatment using mass spectrometry. The lysine concentration in the lysine-free medium was ~19 μM before adding the cells (less than 3% of the normal concentration), and the level dropped to ~1 μM after 3 days of culture (around 0.2% against the control group without lysine depletion). We, hence, defined it as a lysine-free group. The absolute quantification of the amino acids and changes in individual amino acids in the medium was summarized in (Figure 1E and Supplementary Figure S1). Surprisingly, we found that the amino acid levels in the medium were dramatically distorted when cells were cultured in a lysine-depleted medium, suggesting a disruption of amino acid metabolism. Results showed that lysine was constantly absent in the medium before and after treatment. Other than the depletion of lysine, amino acids including glycine, asparagine, alanine, proline, aspartic acid, and glutamic acid were dramatically increased after 3 days of the absence of lysine (Supplementary Figure S1B). The concentrations of arginine, histidine, isoleucine, leucine, methionine, and valine in the medium were not greatly altered after the lysine-free culture, yet the control group consumed these amino acids in the medium. Meanwhile, the levels of glutamine, serine, and cystine were the same as in the control group and had not been altered (Supplementary Figure S1B). These results indicated the induction of a global alteration in amino acid metabolism or excretion via single amino acid deprivation.

### 2.2. Transcriptome Profile of the 3T3-L1 Cells

To unveil the underlying mechanism of how lysine deprivation inhibited adipogenesis, we performed RNA sequencing on cells in the undifferentiated group (Undifferentiated), differentiated group (Differentiated), and the differentiated group treated under lysine-free condition (Lysfree), with n = 3 for each group. An average of 45.32 million raw reads were generated from the samples (ranging from 39.86 million to 54.72 million), while the average clean reads were 44.4 million. From a stringent quality check, >93% of the obtained reads had a quality score of ≥Q30. The heatmap (Figure 2A) and PCA plot (Figure 2B) of all samples proved that the samples were highly consistent within groups and distinctly separated among treatment groups. Adipogenic and lipogenic genes, including FASn, SCD1, SREBF1, and PPARg, were chosen as a quality control against qPCR (Figure 2C). These critical genes that were quantified by RNAseq were in high coherence with the data from the qPCR in magnitude, and all genes were significantly suppressed under lysine deprivation (Figure 2D). The protein level of nuclear transcription factors SREBF1 and PPARg were quantified by Western blot (Supplementary Figure S2), and confirmed the same trend as the data from the RT-PCR and RNAseq.

### 2.3. Comparison between Differentiated and Undifferentiated 3T3-L1 Cells

**Figure 3:**
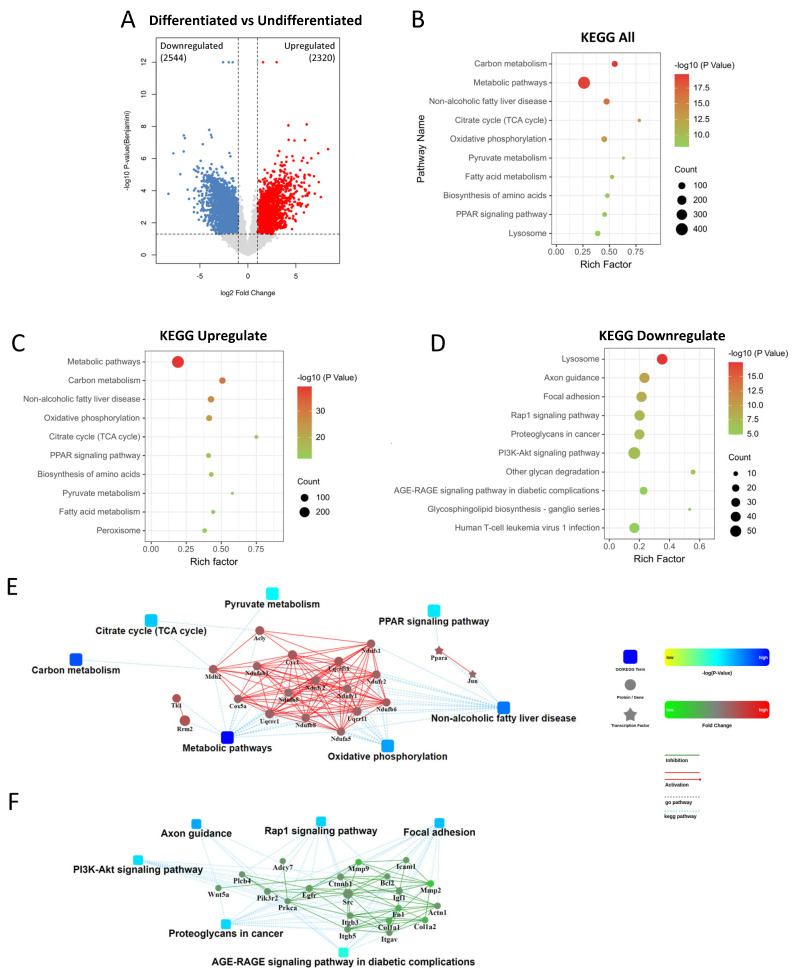
DEGs analysis from the differentiated group against the undifferentiated group. (**A**) Volcano plot presenting the distribution of DEGs with 2320 genes upregulated and 2544 genes downregulated. (**B**) KEGG enrichment of all DEGs. (**C**) KEGG enrichment of the upregulated DEGs. (**D**) KEGG enrichment of the downregulated DEGs. (**E**) PPI analysis of the upregulated pathways. (**F**) PPI analysis of the downregulated pathways.

The differentiated 3T3-L1 cells had 4864 differential expressed genes (DEGs), with 2544 genes downregulated and 2320 genes upregulated (Figure 3A). A KEGG pathways analysis showed that the differentiated cells had the most significant alteration in carbon metabolism and metabolic pathways, and mitochondria-related pathways (Figure 3B). A further analysis of the upregulated and downregulated DEGs individually demonstrated that most of the top altered pathways were upregulated. In the top 10 significantly enriched pathways (Figure 3B), 9 out of 10 were upregulated, and the only downregulated pathway was lysosome. Many of the upregulated pathways were metabolism-related, such as carbon, pyruvate, fatty acid metabolism, and mitochondria-correlated pathways that involved the TCA cycle and oxidative phosphorylation (Figure 3C). As demonstrated by the PPI network, the NADH:ubiquinone oxidoreductase subunit family (Nduf) was highly intercorrelated in several pathways, including Ndufb1, Ndufab1, Ndufb5, Ndufv1, Ndufv2, Ndufb8, Ndufa5, Ndufb6, Ndufc2, and Ndufs1, which were involved in the mitochondria respiratory chain (Figure 3E). The most enriched downregulated pathway was the lysosome pathway (Figure 3D), and surprisingly, PPI network analysis did not reveal any intercorrelation of the lysosome pathway with other pathways; instead, other intercorrelated pathways, including the Rap1 signaling pathway, PI3K-Akt signaling pathway, and AGE-RAGE signaling pathway, were found. Collagen and collagenase-related genes such as matrix metalloproteinase 9 (MMP9), MMP2, collagen type A1 (Colla1), and Colla2 (Figure 3F) were identified. These results suggested that the differentiation of 3T3-L1 dramatically activated metabolic pathways, especially carbon metabolism, which involved mitochondria, while the lysosome pathway was inhibited.

### 2.4. Identification of Lysine-Depletion-Induced Pathways

**Figure 4:**
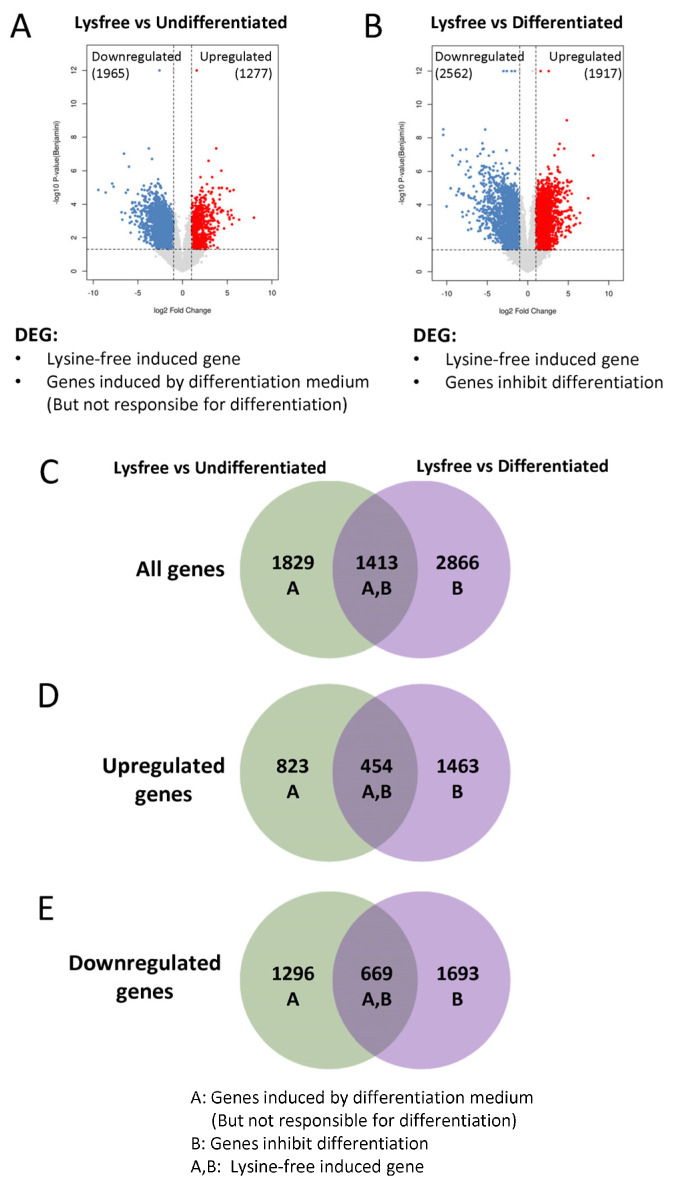
Venn diagram identifying DEGs specifically induced by lysine depletion, genes inhibited adipogenesis, and genes induced by differentiation medium but not critical for adipogenesis. (**A**) Volcano plot presenting the comparison between Lysfree and Undifferentiated groups with 1277 genes upregulated and 1965 genes downregulated. (**B**) Volcano plot presenting the comparison between Lysfree and Differentiated groups with 1917 genes upregulated and 2362 genes downregulated. (**C**–**E**) Venn diagram overlapping (**C**) all DEGs identified, (**D**) all upregulated DEGs identified, and (**E**) all downregulated DEGs identified.

To dissect the lysine-deprivation-induced pathways that inhibited 3T3-L1 adipogenesis, we first compared the lysine-free group against the undifferentiated group (Lysfree vs. Undifferentiated). The DEGs obtained represented lysine-free-induced genes and genes induced by the differentiation medium but not involved in the adipogenesis process (Figure 4A). PPI analysis of the DEGs screened out the ribosome biogenesis pathway and purine and pyrimidine metabolism, which were upregulated, while the PI3K-Akt signaling pathway and ECM–receptor interaction were downregulated (Supplementary Figure S3A). The lysine-free group and the differentiated group were also compared (Lysfree vs. Differentiated). The DEGs obtained represented lysine-free-induced genes and genes that were critical for blocking adipogenesis (Figure 4B). PPI analysis on DEGs demonstrated that genes were downregulated in many metabolic pathways, especially carbon metabolism and mitochondria-related pathways such as the TCA cycle and oxidative phosphorylation (Supplementary Figure S3B). Moreover, interleukin 6 (IL6) was identified to have been significantly and dramatically upregulated in comparison with the undifferentiated or the differentiated groups (Supplementary Figure S3C). The DEGs from the two comparisons were then overlapped by Venn diagrams (Figure 4C), and the overlapped DEGs were considered as lysine-free-induced genes. Other than overlapping the DEGs of the two groups, we also individually analyzed the upregulated (Figure 4D) and downregulated DEGs (Figure 4E) to screen out the lysine-free enhanced and suppressed pathways, respectively.

**Figure 5:**
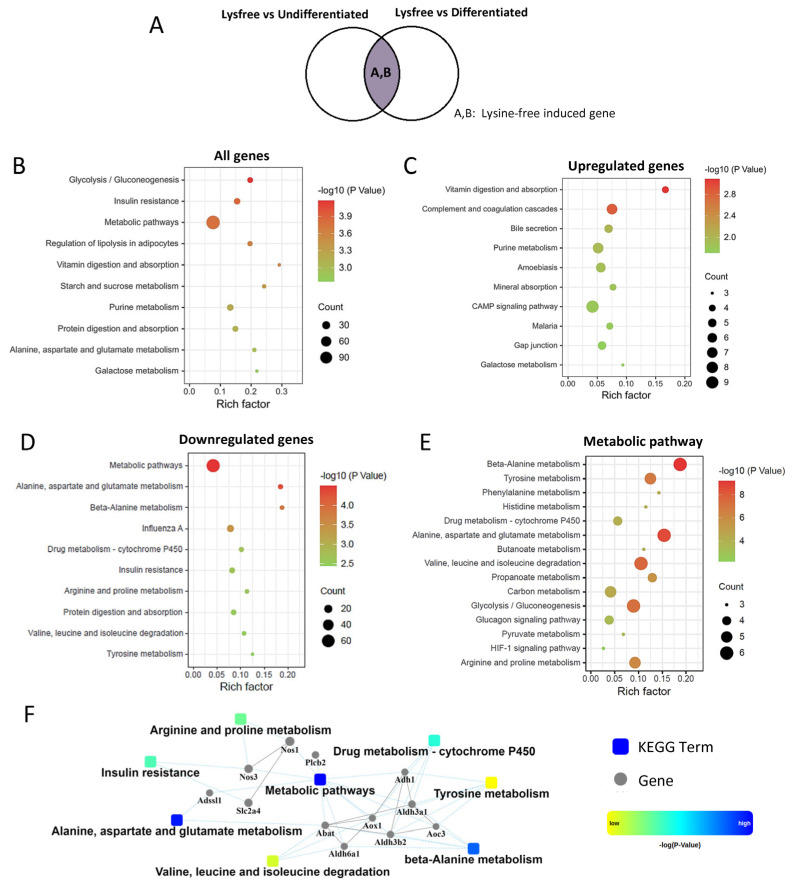
Lysine depletion dramatically downregulated amino acids and carbon metabolic pathways. (**A**) Venn diagram showing the portion of DEGs analyzed. (**B**–**D**) KEGG enrichment of DEGs induced by lysine-auxotroph: (**B**) all DEGs, (**C**) the upregulated DEGs, and (**D**) the downregulated DEGs. (**E**) KEGG enrichment of the downregulated DEGs from the metabolic pathways. (**F**) PPI analysis of the downregulated metabolic pathways.

The overlapped DEGs representing lysine-free-induced genes (Figure 5A) are presented in Figure 5. KEGG analysis demonstrated that the most enriched pathway was vitamin digestion and absorption, while the highest gene counts were metabolic pathways (Figure 5B). In terms of upregulated genes, vitamin digestion and absorption was the top enriched pathway (Figure 5C). For downregulated DEGs, the most enriched pathway was alanine, aspartate, and glutamate metabolism, and the highest gene counts were the metabolic pathway (Figure 5D). We further dissected the 60 genes in metabolic pathways using Metascape (https://metascape.org/gp/index.html#/main/step1, accessed on 1 February 2023). Most of the metabolic pathways were found to be correlated with amino acid metabolism (particularly beta-alanine, tyrosine, phenylalanine, histidine, alanine, aspartate and glutamate, valine, leucine, and isoleucine metabolic pathways), and carbon metabolism pathways (including glycolysis/gluconeogenesis, glucagon signaling, and pyruvate metabolism) (Figure 5E). These results clearly showed that lysine deprivation specifically and strongly suppressed amino acids and carbon metabolism pathways in differentiated 3T3-L1 cells. PPI network analysis of the downregulated genes screened out dehydrogenase (including ADH1, Aldh6a1, Aldh3a1, and Aldh3b2), nitric oxide synthase (Nos1 and Nos3), and Slc2a4, an insulin-sensitive glucose transporter (known as glucose transporter 4, GLUT4) (Figure 5F).

### 2.5. Genes Critical for Inhibition of 3T3-L1 Cell Differentiation and Adipogenesis

**Figure 6:**
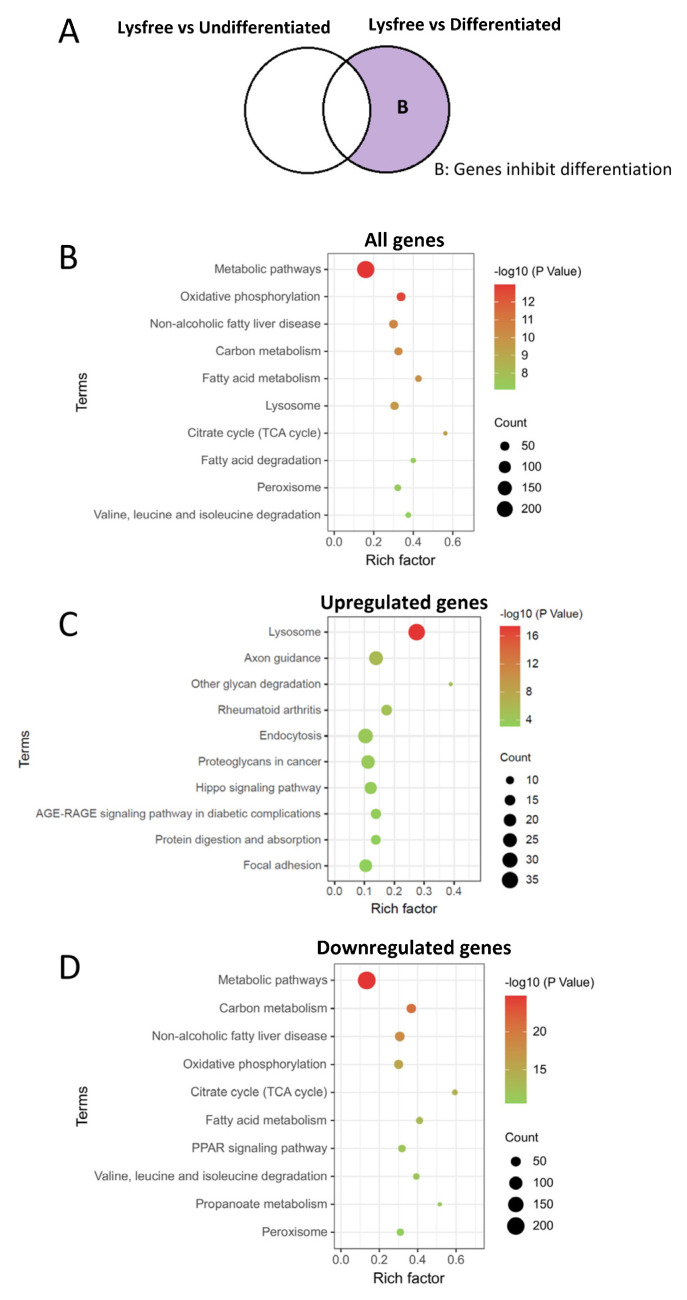
Suppression of metabolic pathways and upregulation of lysosome pathway were identified as critical transcriptomic changes for the inhibition of the adipogenesis process. (**A**) Venn diagram showing the portion of DEGs analyzed. (**B**–**D**) KEGG enrichment of DEGs, (**B**) all DEGs, (**C**) the upregulated DEGs, and (**D**) the downregulated DEGs.

To investigate the genes that were essential for the suppression of adipogenesis, DEGs between the Lysine-free and Differentiated groups, excluding the lysine-free-induced genes, were enriched with KEGG (Figure 6A). Interestingly, the results were opposite in the DEGs between the differentiated and undifferentiated groups (Figure 3). Eight out of the top ten enriched pathways were found to have been downregulated, and the majority of them were metabolic pathways, especially the mitochondria-related pathways, including carbon metabolism, oxidative phosphorylation, TCA cycle, and fatty acid metabolism (Figure 6D). To verify the functional impact in the suppression of mitochondrial TCA cycle and oxidative phosphorylation, we examined the mitochondrial ATP synthesis on 3T3-L1 cells using Agilent Seahorse XF ATP Real-Time rate assay. Concomitant with the transcriptomic data, a significant reduction in ATP synthesis was detected (Supplementary Figure S4), proving the consistency between transcriptomic and cellular functional changes. The most upregulated pathway was the lysosome pathway (Figure 6C). These results provided evidence to support the critical role of carbon metabolism, mitochondria function, and lysosome in the adipogenesis process.

### 2.6. Pathways That Could Be Altered by Differentiation Medium but Not Critical for the Adipogenesis Process

**Figure 7:**
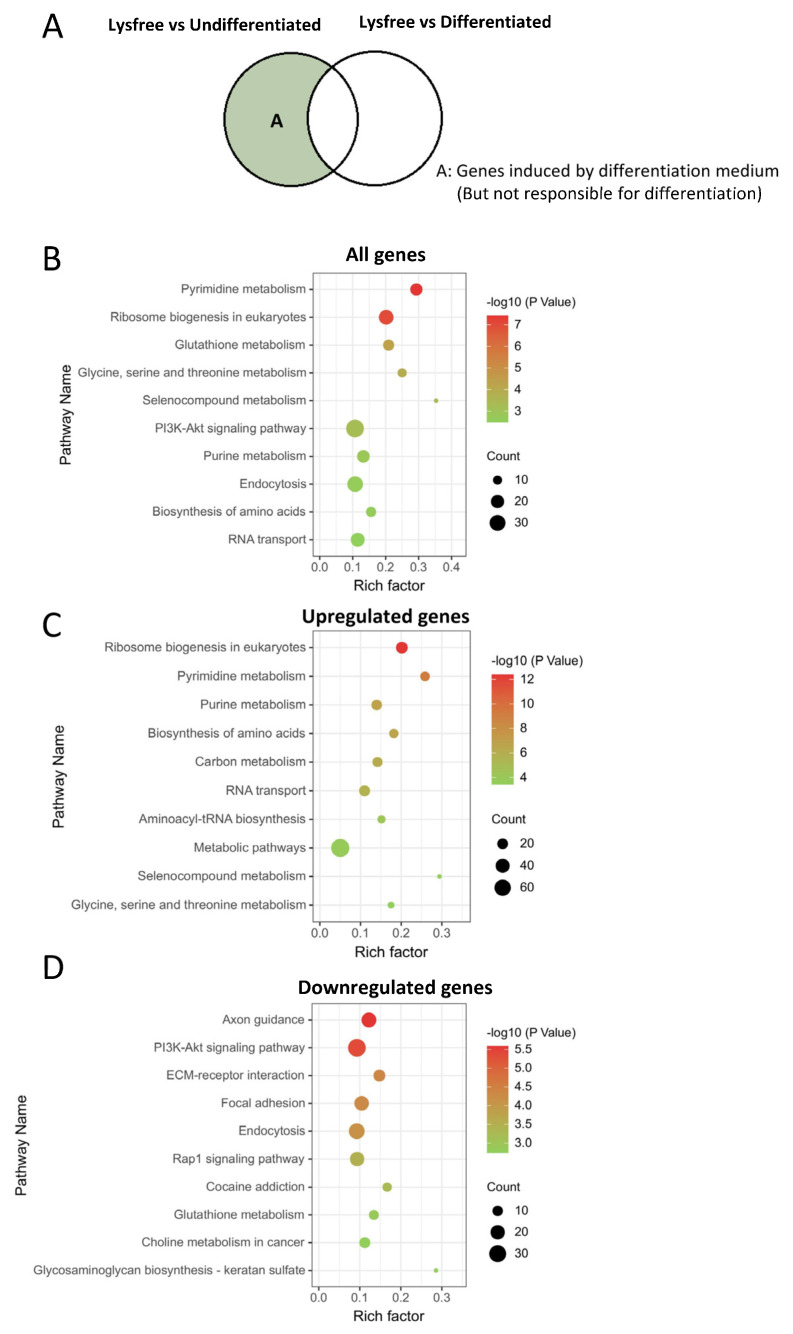
Identification of pathways that did not play any functional roles in adipogenesis but were induced during cell differentiation. (**A**) Venn diagram showing the portion of DEGs analyzed. (**B**–**D**) KEGG enrichment of DEGs, (**B**) all DEGs, (**C**) the upregulated DEGs, and (**D**) the downregulated DEGs.

We also examined the pathways that were altered by the differentiation medium but were not related to adipogenesis by enriching the DEGs between the Lysine-free and Undifferentiated groups, excluding lysine-free-induced genes (Figure 7A). The ribosome biogenesis, pyrimidine, and purine metabolism pathway was the most enriched upregulated pathway (Figure 7B,C), and it was also identified to be upregulated in the Lysine-free group against the Undifferentiated group (Supplementary Figure S3A), suggesting that these pathways were upregulated accompanied with cell differentiation medium but they were not critical for 3T3-L1 cells adipogenesis. We also found enriched downregulated pathways such as the Rap1 signaling pathway, the PI3K-Akt signaling pathway, and ECM–receptor interaction (Figure 7D) that were the same as the downregulated KEGG pathways in comparing the Differentiated and Undifferentiated group (Figure 3D), proving that these pathways were not important for 3T3-L1 adipogenesis, just for the cellular response to differentiation medium.

### 2.7. Suppression of Adipogenesis Is Not Limited to Lysine Depletion but Also Applied to Methionine and Cystine Deprivation Partially via IL6 Overexpression

**Figure 8:**
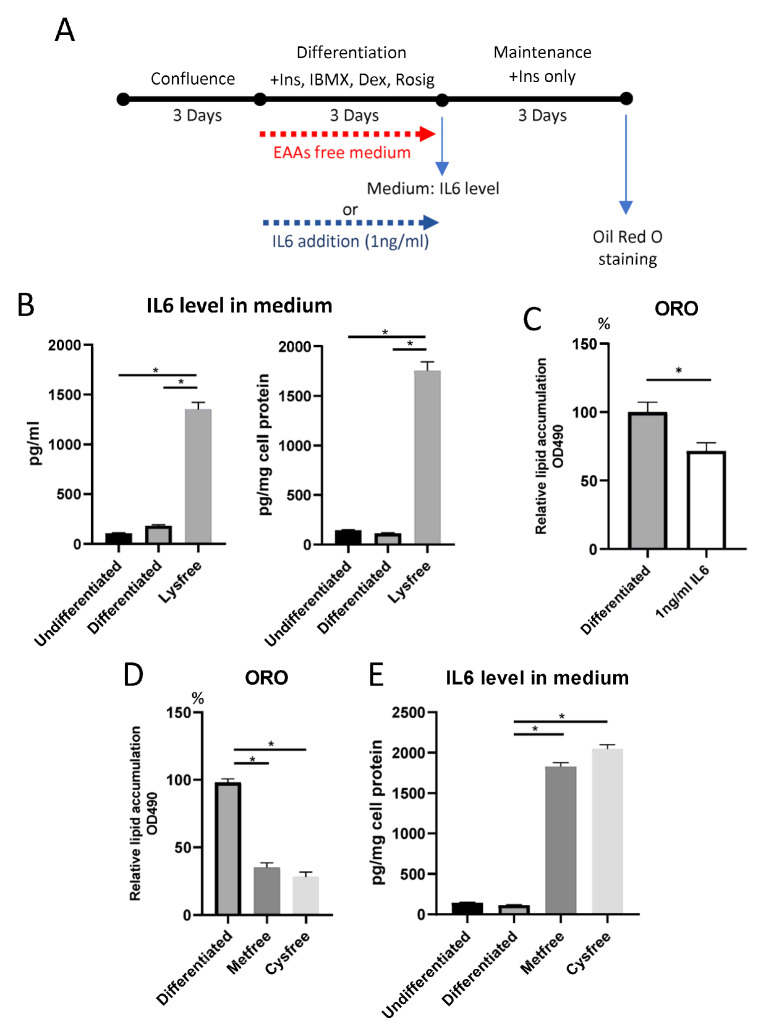
IL6 level in the medium was significantly increased in 3T3-L1 cells cultured under amino acid depletion medium for 3 days. (**A**) The schematic diagram demonstrated the experimental design. (**B**) IL6 level in medium raised in cells cultured under lysine-free medium. Data presented as pg/mL (left) and pg/mg cell protein (right). (**C**) Adipogenesis was partially suppressed by treatment with 1 ng/mL IL6 in the medium during the differentiation period. Data are expressed as means ± SE with n = 4 in each condition * *p* < 0.05, Mann–Whitney U test. (**D**) Methionine and Cystine depletion (Metfree and Cysfree) suppressed the adipogenesis of 3T3-L1 cells. (**E**) IL6 concentration in the medium was increased in cells cultured under Metfree and Cysfree environments. For (**B**,**D**,**E**), data are expressed as means ± SE with n = 3 in each condition * *p* < 0.05, one-way ANOVA followed by the Bonferroni test.

We have identified that IL6 was overexpressed in cells exposed to a lysine-free environment (Supplementary Figure S3). To explore whether IL6 could be one of the mechanisms for the suppression of adipogenesis, we first examined the IL6 level in the culture medium. Results showed that the medium IL6 level was dramatically increased 8-fold (Figure 8B) in the medium when cells were cultured under lysine starvation, and the upregulation was inversely proportional to medium lysine concentration (Supplementary Figure S5A). We found that such overexpression of IL6 is reversible after refeeding the cells with 800 μM lysine (Supplementary Figure S5B,C). To test whether such an increase in IL6 could contribute to the inhibition of adipogenesis, we added an equivalent amount of IL6 (1 ng/mL) in the medium during differentiation and examined the adiposity by Oil-Red O staining. We found that the cellular triglyceride content was partially suppressed (Figure 8C). We also tested whether such phenomena can be observed in other amino-acid-depleted conditions. Other than lysine depletion, a medium lack of essential amino acid methionine and cysteine individually could also suppress the adipogenesis process on 3T3-L1 cells, the same as lysine deprivation (Figure 8D). Coincidentally, the IL6 level in the medium was significantly upregulated in an amino acid depletion environment (Figure 8E), suggesting that the phenomenon is not just lysine-specific but also applicable to other essential amino acids.

## 3. Discussion

The current descriptive study is consistent with previous findings that showed that mRNA expressions of PPARg (Figure 2C,D) and C/EBPa (Supplementary Figure S6A) were inhibited in cells cultured in a low-lysine environment [27]. On the other hand, gene expressions of C/EBPb and C/EBPd were not downregulated by lysine deprivation in the culture medium (Supplementary Figure S6A). These results indicate that lysine depletion in the culture medium inhibited the differentiation of 3T3-L1 preadipocytes mainly by inhibiting the mRNA expressions of PPARg and C/EBPa. RNA-seq data comparing differentiated and undifferentiated 3T3-L1 cells were reported by Sun et al. [28]. Although our experimental design was not exactly the same, we found that our findings match their reported data. Trpv4, Trpm4, and Trpm5 were significantly downregulated, while Trpv1, Trpv2, and Trpc1 significantly increased in differentiated adipocytes (Supplementary Figure S6B,C).

To examine whether lysine starvation could enhance the cellular lipid breakdown, we checked the transcription of the three major lipases, adipose triglyceride lipase (ATGL), hormone-sensitive lipase (HSL), and monoacylglycerol lipase (MGL), in the lipolysis pathway, which breaks triglyceride into glycerol and free fatty acid. Surprisingly, all three genes were significantly suppressed under lysine depletion (Supplementary Figure S7). Results suggest that lysine depletion could inhibit adipogenesis but not enhance lipolysis. Lipid metabolism is overall impaired in lysine-deficient conditions. The alteration of lipid metabolism by amino acid depletion has been reported in animal models. It has been shown that a deficiency in BCAAs including leucine, isoleucine, and valine individually can stimulate lipolysis in white adipose tissue [29,30]. Lysine deprivation, however, did not demonstrate such a change in lipolysis [11]. The controversy between the current and the reported in vivo data on lipolysis suggests that amino acid depletion in vivo is much more complex than in in vitro models, particularly the degree of amino acid depletion in the bloodstream and in the cells.

The effect of lysine depletion on body composition in vivo is also controversial. In rats, lysine depletion can reduce body weight, fat mass, and lean mass [2,31]. Interestingly, pigs fed a lysine-depletion diet demonstrated a lower relative proportion of lean components; however, higher carcass fatty components were obtained, together with increased lipogenic genes [32]. Since lysine is an essential amino acid, long-term lysine depletion can be harmful, and currently, no human data are available.

3T3-L1 cells could be the target cells for IL6. We identified that IL6 gene expression was significantly upregulated in a lysine-free environment (Supplementary Figure S3C). Coherent with the transcriptomic data, we found that the IL6 level in the culture medium was dramatically increased after treatment with a lysine depletion medium for 3 days (Figure 8B). To test whether the increased IL6 level could suppress the adipogenesis of 3T3-L1 cells, we added an equivalent amount of IL6 (1 ng/mL) in the medium during differentiation and examined the adiposity of cells. Surprisingly, the differentiation process was partially inhibited, suggesting that IL6 upregulation is one of the mechanisms for the lysine depletion environment to suppress adipogenesis (Figure 8C). IL6 has emerged as a crucial cytokine involved in metabolism regulation. It was reported that 3T3-L1 cells treated with IL6 displayed fewer lipids [33]. Furthermore, IL6 treatment led to decreased mitochondrial membrane potential, decreased cellular ATP production, and increased intracellular ROS levels. Consistent with previously published findings, we found that peroxisome proliferator-activated receptor gamma coactivator 1 alpha (PPARGC1a) and nuclear respiratory factor 1 (NRF1) expression levels were markedly increased, an upregulation triggered by IL6 (Supplementary Figure S8A) [33]. Additionally, the gene transcription of insulin receptor substrate 1 (IRS-1), solute carrier family 2 member 4 (called Slc2a4 or glucose transport 4) (Supplementary Figure S8B), and PPARg (Figure 2C,D) were found to be significantly inhibited in cells under lysine depletion, a phenomenon that was also observed in 3T3-L1 cells treated with exogenous IL6 [34]. IRS-1 is responsible for insulin signaling, Slc2a4 is essential for the cellular glucose uptake, and PPARg is the key gene for adipogenesis. The expression of all these genes is essential for the formation of lipid droplets and the maturation of adipocytes. In animal models, during obesity development, IL6 production in adipose tissues is consistently elevated, especially in insulin-resistance adipocytes [35,36]. Such IL6 overexpression reduced subcutaneous adipocyte adipogenesis capacity with suppressed PPARg and C/EBPa expression [35], which is similar to our in vitro findings. However, how lysine, methionine, and cysteine depletion regulate IL6 levels is still unknown and worth investigating. We hypothesize that oxidative stress may be associated with IL6 overexpression. Lysine level was found to be correlated with antioxidant capacity [37]. Deficiencies in methionine and cysteine can affect the transsulfuration pathway and greatly reduce antioxidant glutathione production [38]. It has been reported that total glutathione levels were decreased by 42% in melanoma cells grown without methionine, and by 95% in cells grown without cysteine [39]. Importantly, oxidative stress can induce insulin resistance in adipocytes and dramatically increase IL6 secretion in 3T3-L1 cells 3-fold [40,41].

Amino acid deprivation increases energy expenditure and reduces food intake and fat mass, primarily through the regulation of the general control nonderepressible 2 (GCN2) and mammalian target of rapamycin (mTOR) signaling. GCN2 and mTORC1 signaling pathways have been extensively studied for their regulation by amino acids, especially in the control of translation. GCN2 activated by uncharged tRNA during scarcity of amino acid can phosphorylate the α-subunit of eukaryotic initiation factor 2 (eIF2a) [42,43] and promote the translation of certain mRNAs by activating transcription factor 4 (ATF4) [44], which plays a key role in the adaptation of the cells to the lack of amino acids [45]. Surprisingly, we did not find any significant changes in GCN2, eIF2a, or downstream ATF4 expression. This phenomenon may indicate that 3T3-L1 cells cultured under a lysine deprivation medium may not really be under intracellular amino acid shortage but have other cellular changes to overcome the amino acid scarcity. We found that the lysosome pathway was strongly upregulated in both undifferentiated cells (Figure 3D and Supplementary Figure S9) and lysine-free-treated cells (Figure 6C). Lysosomes are an important component of the inner membrane system and participate in numerous cell biological processes, such as macromolecular degradation, intracellular pathogen destruction, plasma membrane repair, exosome release, and apoptosis [46]. The increase in lysosome might break down unused intracellular protein or damaged organelles to recycle some amino acids, which could provide an extra source of lysine for cell survival. Interestingly, we found that some lysosomal proteases such as Legumain (LGMN), tripeptidyl peptidase 1 (TPP1), and Cathepsin (CTS) (Supplementary Figure S9B,C) were upregulated, which could be one of the reasons for the increased amino acids in the medium, as described in Figure 1E and Supplementary Figure S1. Another lysosomal amino-acid-sensing pathway is the mammalian target of rapamycin (mTOR) pathway; however, no changes in the whole pathway, including amino-acid-sensing protein CASTOR1, regulatory-associated protein of mTOR (Raptor), mTOR, downstream S6 Kinase 1 (S6K1), and translation initiation factor 4E binding protein (4EBP), were observed. The activation of mTOR signaling involved the phosphorylation of proteins instead of gene expression. Further proteomic studies are required to confirm the mTOR signaling pathway and other significant DEGs identified.

Various beneficial effects of dietary amino acid restriction, including anti-obesity, anticancer, and anti-aging effects in both obese patients and animal models, have been proven [47,48]. For example, it has been shown that dietary methionine restriction could prevent obesity. Methionine restriction by 80% decreased fat mass and body weight, primarily through fibroblast growth factor 21 (FGF-21) signaling [49,50]. More recently, it was shown that the oral intake of methioninase (METase), an enzyme catabolizing methionine, could significantly prevent the development of obesity symptoms and could be used as an anticancer treatment, demonstrating the potential for using amino-acid-depleting enzymes as therapeutic agents [51,52]. Moreover, the injection of pegylated arginine deaminase (ADI-PEG), an arginine-depleting enzyme, also demonstrated similar effects [53]. L-lysine oxidase, an enzyme that converts L-lysine to 6-amino-2-oxohexanoate, NH_3_, and H_2_O_2_, has been applied in in vivo in cancer treatment. L-lysine oxidase treatment completely inhibited the growth of mouse leukemic cells in vitro [54]. An intravenous injection of L-lysine oxidase to mice suffering from leukemia increased their average lifespan compared with the control animals [55]. We predicted that, like METase and ADI, L-lysine oxidase could be another candidate enzyme drug to combat obesity and aging. In animal studies, dietary lysine deprivation has been proven to decrease food intake and increase energy expenditure [6] mediated by enhanced serotonin release from the amygdala in rats [56].

## 4. Materials and Methods

### 4.1. Cell Culture

3T3-L1 cells were obtained from ATCC. They were maintained in DMEM (Gibco, New York, NY, USA, #12800017) with 10 % BCS (Gibco, #16170078) at confluence for 3 days. The cells were then stimulated with a Differentiation medium (arginine and lysine-free DMEM (Gibco, #88364) supplemented with 10%FBS (Gibco, #26140-079), 800 μM lysine (Sigma, Marlborough, MA, USA), 400 μM arginine (Sigma), 1 μM dexamethasone (DEX, Sigma), 0.5 mM 3-isobutyl-1-methylxanthine (IBMX, Sigma), 1 μM rosiglitazone (Rosig) (Cayman, Ann Arbor, MI, USA), and 10 μg/mL insulin (Sigma)) to induce adipocyte differentiation. After differentiation, the medium was replaced with Maintenance medium (DMEM (Gibco, #12800017) supplemented with 10 % FBS (Gibco, #26140-079) and 10 μg/mL insulin) for 3 days. For lysine-free treatment, confluent pre-adipocytes were treated with a Differentiation medium without lysine addition during the differentiation, and the lysine-free treatment was stopped after the differentiation period. For the dose-dependent study, various concentrations of lysine (as indicated in Figure 1D) were supplemented during differentiation. For methionine and cystine depletion, the DMEM in the Differentiation medium was substituted with DMEM, no glutamine, no methionine, and no cystine (Gibco, #21013-024), supplemented with 4 mM glutamine (Sigma) and either 200 μM methionine (Sigma) or 200 μM cysteine (Sigma). Cells and medium were collected for RNA extraction and IL6 quantification after differentiation. Medium IL6 concentration was determined by a mouse IL6 ELISA assay kit (Invitrogen, Waltham, MA, USA). Cell viability was determined using MTT assay. Oil-Red O staining was performed after the maintenance period to check the cell adiposity.

To examine the effect of IL6 on 3T3-L1 cell adipogenesis, cells were differentiated in DMEM (Gibco, #12800017) supplemented with 10%FBS (Gibco, #26140-079), 1 μM dexamethasone, 0.5 mM IBMX, 1 μM Rosig, and 10 μg/mL insulin with or without the addition of 1 ng/mL IL6 (Sigma) for 3 days. To test whether the upregulation of IL6 is reversible, cells were first differentiated for 2 days and refed with 800 uM lysine for 1 day.

### 4.2. RNA Extraction, cDNA Synthesis, and RNA Sequencing

The total RNA was isolated using the total RNA extraction kit (Favorgen, Pingtung, Taiwan) following the manufacturer’s instructions. Both qualities and quantities were measured using Bioanalyzer 2200 (Agilent Technologies, Santa Clara, CA, USA). The sequencing library of each sample was built using poly A enrichment preparation by the sequencing service provider Novogene (Beijing, China). Sequencing was performed using the NovaSeq (Beijing, China) PE150 platform with 150 bp paired-end reads generated. Low-quality reads (Qscore > 50% of the read) and reads with adaptors were removed to obtain clean data. The clean data were mapped to the mouse genome version 10 (mm10) using the HISAT2 algorithm with default parameters. RNAseq raw data are available in GEO database with accession number GSE223846.

### 4.3. Differentially Expressed GENES (DEG) Analysis

DESeq2 algorithm on OmicsBeam (http://omicsbean.cn/, accessed on 1 February 2023) was used to identify the differentially expressed genes (DEGs) in various treatment groups. DEGs were defined as showing a ≥2-fold change and padj < 0.05. Raw count values greater than 0 in any group were used. Principal component analysis (PCA) and hierarchical clustering were carried out to assess the variability in and repeatability of samples using the normalized RNA-Seq read counts. A volcano plot was used to visualize the overall distribution of DEGs.

### 4.4. Functional Annotation Analysis

Pathway analysis was used to highlight the significant pathways of the DEGs using the Kyoto Encyclopedia of Genes and Genomes database (KEGG, http://www.genome.jp/kegg/, accessed on 1 February 2023) [57], which stores information on how molecules and genes are networked, for pathway mapping. Pathways showing adjusted *p*-values < 0.05 were selected.

### 4.5. Integration of Protein–Protein Interaction (PPI) Network and Module Analysis

The Search Tool for the Retrieval of Interacting Genes (STRING) (http://string-db.org, accessed on 1 February 2023) [58] is an online tool designed for evaluating the differentially expressed mRNA-encoded proteins and PPI. The OmicsBean was used to construct a protein interaction relationship network and analyze the interaction relationships of differentially expressed candidate genes based on the STRING analysis results.

### 4.6. Real-Time PCR Analysis

Real-time PCR was performed using a high-capacity cDNA Reverse Transcription Kit (Applied Biosystem) to synthesize cDNA. Real-time PCR was performed on Real-Time System QS7 (Applied Biosystem, Waltham, MA, USA) with Powerup SYBR (Applied Biosystem) according to the manufacturer’s instructions. TATA box binding proteins (Tbp) were used as housekeeping genes. The (2−ΔΔCt) method was used to calculate the gene expression levels. The primer sequence of target genes and internal control Tbp are summarized in Table 1.

### 4.7. Oil-Red O (ORO) Staining

To examine the lipid droplets, ORO staining was performed. Briefly, the adipocytes were washed twice with PBS. Cells were then fixed with 10% formaldehyde for 15 min at room temperature, followed by PBS wash. Then, the cells were stained with freshly prepared ORO working solution in 60% isopropanol for 20 min. After staining, the images were captured with an Axiovert 40 CFL Zeiss microscope (Carl Zeiss, Baden-Württemberg, Germany) at a magnification of 40×. The intracellular lipid content was quantified by measuring the absorbance at 490 nm.

### 4.8. Medium Amino Acid Analysis

To determine the concentration of amino acids in the medium, Kairos Amino Acid Kit (Waters Corporation, Milford, MA, USA) was used. Briefly, 50 µL samples were mixed with 50 µL of Internal Standard and 50 µL of water and centrifuged for 15 min at 9000× *g*. Then, 10 microliter supernatant was collected and mixed with 70 µL of Borate buffer in a maximum recovery vial. The solution was derivatized by 20 µL AccQ-Tag reagent and then heated for 10 min at 55 °C. After the derivatization, 2 µL samples were analyzed by Agilent 6460 Triple Quadrupole liquid chromatography/mass spectrometry (Agilent Technologies, CA, USA).

### 4.9. Western Blotting

Twenty milligrams of cell protein lysate was resolved by SDS-PAGE and transferred to nitrocellulose membranes (Bio-Rad, Hercules, CA, USA) following standard protocol. Primary and secondary antibodies: PPARg (CST, Fall River, MA, USA, #2435), SREBF1 (Thermofisher, Waltham, MA, USA, #MA511683), GAPDH (CST, #2118), and HRP goat anti-rabbit secondary antibody (CST, #7074). The bands were captured using X-ray film (Fujifilm, Tokyo, Japan) exposed by chemiluminescent HRP substrate (Millipore, Burlington, MA, USA). 

### 4.10. Agilent Seahorse XF ATP Real-Time Rate Assay

3T3-L1 cells were cultured in XFe96 cell culture microplate (Agilent) following previously described with the 3 groups, undifferentiated, differentiated, and lysine-free. After the differentiation stage, cells were subject to Agilent Seahorse XF ATP Real-Time rate assay following manufacturer’s protocol. In brief, basal oxygen consumption rate (OCR) was first monitored followed by addition of 15 μM Oligomycin to block the mitochondrial ATP synthase. The reduction in OCR representing the mitochondrial ATP synthesis was calculated using software Wave (Agilent Technologies, CA, USA).

## 5. Conclusions

The current research described the transcriptomic changes in 3T3-L1 cells in the absence of lysine in a culture medium. Other than traditionally identified pathways, we found that mitochondria and lysosome pathways were critical for adipocyte differentiation. Cells cultured under a lysine-depleted environment triggered the disruption of amino acid and carbon metabolism. We also identified that IL6 was overexpression in lysine-free conditions and can partially suppress the adipogenesis process in 3T3-L1 cells. Functional and proteomic studies are worth conducting to further confirm the findings in the current research. Our new findings shed light on the importance of manipulating amino acids as therapeutic targets against obesity and provide evidence to support the use of lysine-depleting enzymes for therapy.

## Figures and Tables

**Table 1:** Primer sequence for qPCR.

Gene	Forward	Reverse
FASn	CCCAGCGGTAGAGAATAGCA	GGGTCCACTAAACTGAGCCT
SCD1	TCCAACTCATGTGCCTCTGT	AACAACCAACCCTCGCATTC
SREBF1	TTGTTCCTTTGCCTTCCAGC	GATGCCGACCAGATTCCCTA
PPARg	TGACAGACCTCAGGCAGATC	AGAAGGAACACGTTGTCAGC
IL6	TCCTTCCTACCCCAATTTCCA	GTCCACAAACTGATATGCTTAGG
Tbp	TGCTGTTGGTGATTGTTGGT	ACTGGGAAGGCGGAATGTAT

## Data Availability

RNAseq raw data are available in the GEO database with access number GSE223846.

## Supplementary Materials

The following supporting information can be downloaded at: https://www.mdpi.com/article/10.3390/ijms24119402/s1.
