# Supplementary material for: Lysine Deprivation Suppresses Adipogenesis in 3T3-L1 Cells: A Transcriptome Analysis
Source: Int J Mol Sci. 2023 May 28;24(11):9402. doi: 10.3390/ijms24119402 (PMC10253796; doi:10.3390/ijms24119402)

Supplementary Figures and Table
Lysine deprivation suppresses adipogenesis in 3T3-L1 cells: A transcriptome analysis

Leo M.Y. Lee, Z. Q. Lin, L. X. Zheng, Y. F. Tu, Y. H. So, X. H. Zheng, T.J. Feng, X. Y. Wang, W. T. Wong, Y.C. Leung

Supplementary Figure S1. Absolution quantification of the amino acids level in medium. Three samples from an individual experiment were pooled and subjected to quantification. (A) Table summarized the quantified amino acids level in medium. (B) Graphs demonstrating the change of individual amino acids levels in medium before and after treatment.

A

	Amino acid concentration (uM)			
	Before treatment		After treatment	
sample	Differentiated	Lysine Free	Differentiated	Lysine free
Histidine	247.3	190.2	127.0	226.3
Asparagine	1.0	1.0	40.1	85.2
Arginine	300.4	300.4	122.9	292.0
Serine	369.4	398.3	23.9	148.0
Glutamine	2482.1	2437.5	456.1	286.2
Glycine	425.7	461.7	849.9	1208.1
Aspartic acid	9.8	10.5	18.9	54.8
Glutamic Acid	76.6	84.0	141.2	400.6
Threonine	665.0	718.3	548.9	816.8
Alanine	80.4	87.2	624.1	1293.4
Proline	17.5	19.2	117.8	449.0
Lysine	627.7	18.6	440.6	1.2
Cystine	383.5	457.3	165.1	249.7
Tyrosine	336.3	364.3	276.6	429.1
Methionine	151.9	166.5	86.8	174.7
Valine	663.2	702.7	406.8	662.4
Isoleucine	672.5	726.8	228.2	562.6
Leucine	688.3	744.9	233.7	574.5
Phenylalanine	340.1	368.6	264.0	430.6
Tryptophan	65.9	70.4	45.0	87.7
Beta Alanine	9.4	10.7	9.9	22.2

B

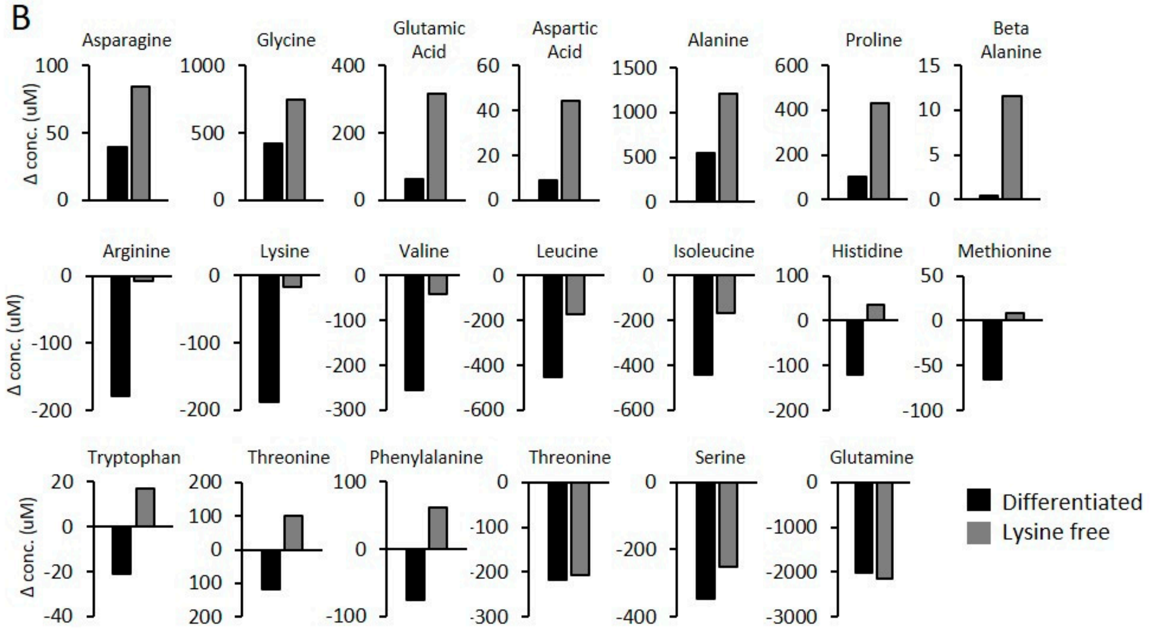

Supplementary Figure S2. Protein level of the key nuclear transcription factors PPARg and SREBF1 were suppressed under lysine-free treatment. (A) The schematic diagram demonstrated the experimental design for protein analysis using western blotting (WB). (B) WB image of PPARg, SREBF1 and GAPDH in differentiated cells and cells under lysine-free medium.

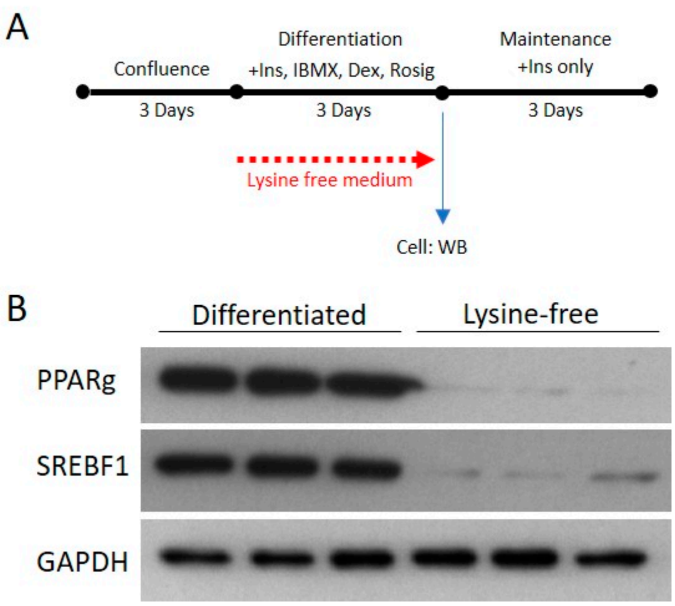

Supplementary Figure S3. Lysine deprivation triggered IL6 upregulation. (A) PPI analysis of all DEGs from the KEGG enriched pathways in Lysfree against the Undifferentiated group. (B) PPI analysis of all DEGs from the KEGG enriched pathways in Lysfree against Differentiated group. (C) Gene expression level of the IL6 in fpkm from RNAseq analysis. Data were expressed as means ± SE with n=3 in each condition. *** $p < 0.001$ using the One-way ANOVA followed by the Bonferroni test.

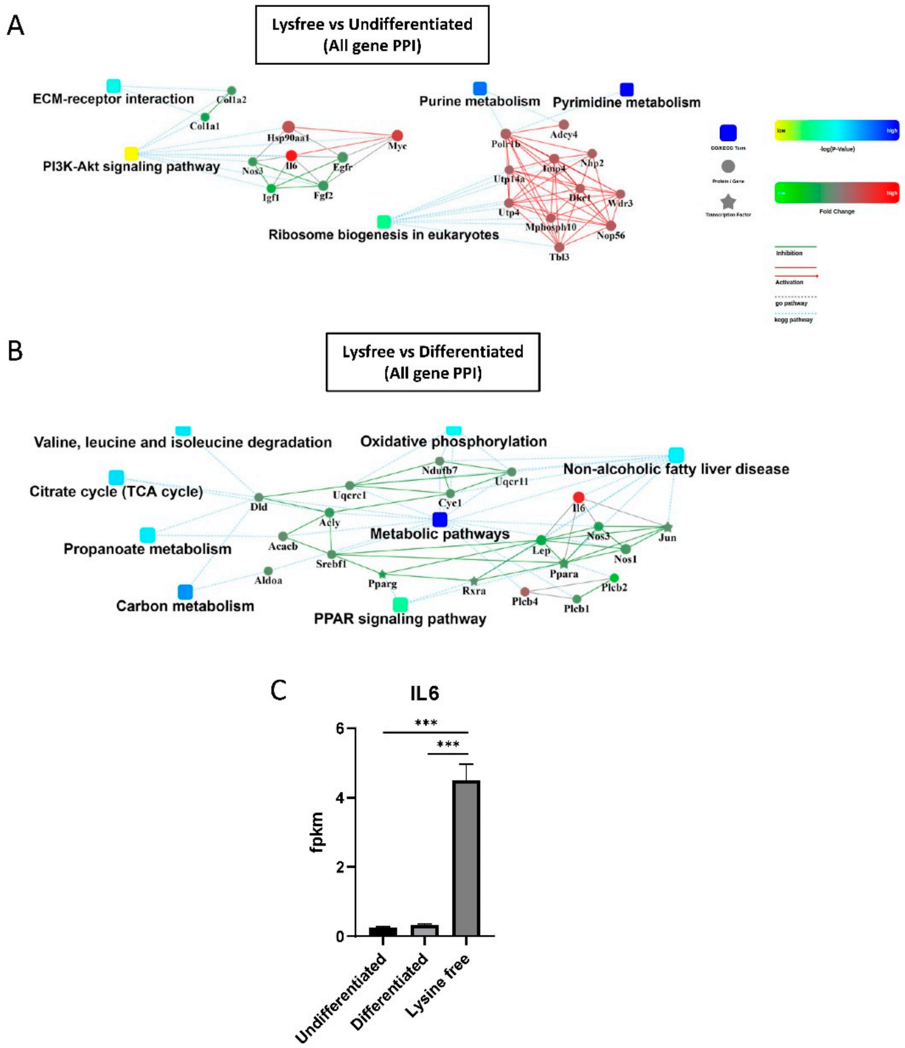

Supplementary Figure S4. Mitochondrial ATP synthesis was inhibited in 3T3-L1 cells cultured under lysine-free medium. ATP synthesizing rate calculated from the change in oxygen consumption rate after adding mitochondria ATP synthase inhibitor, oligomycin. Data were expressed as means ± SE with n=6 in each condition. $**p < 0.01$ and $***p < 0.001$ using the One-way ANOVA followed by the Bonferroni test.

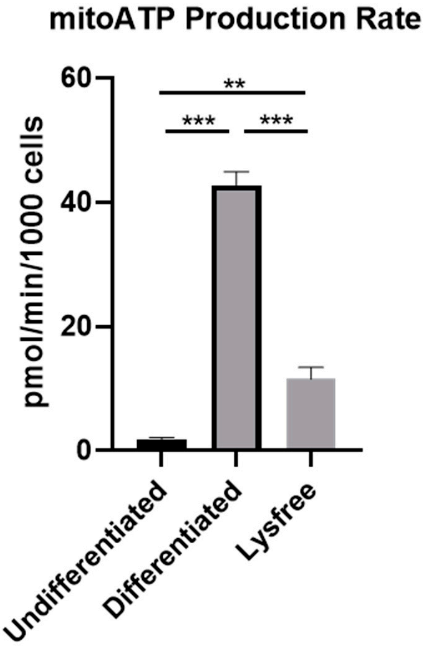

Supplementary Figure S5. The up-regulation of IL6 expression is dose-dependent and reversible upon lysine refeeding. (A) The expression level of IL6 is inversely proportional to medium lysine concentration. Correlation was analyzed using Pearson correlation test. (B) Schematic diagram for experimental design of the lysine refeeding. (C) Addition of 800uM lysine in medium can reversed the over-expression of IL6 in 3T3-L1 cells. Data were expressed as means ± SE with n=3 in each condition. ***p < 0.001 using the One-way ANOVA followed by the Bonferroni test.

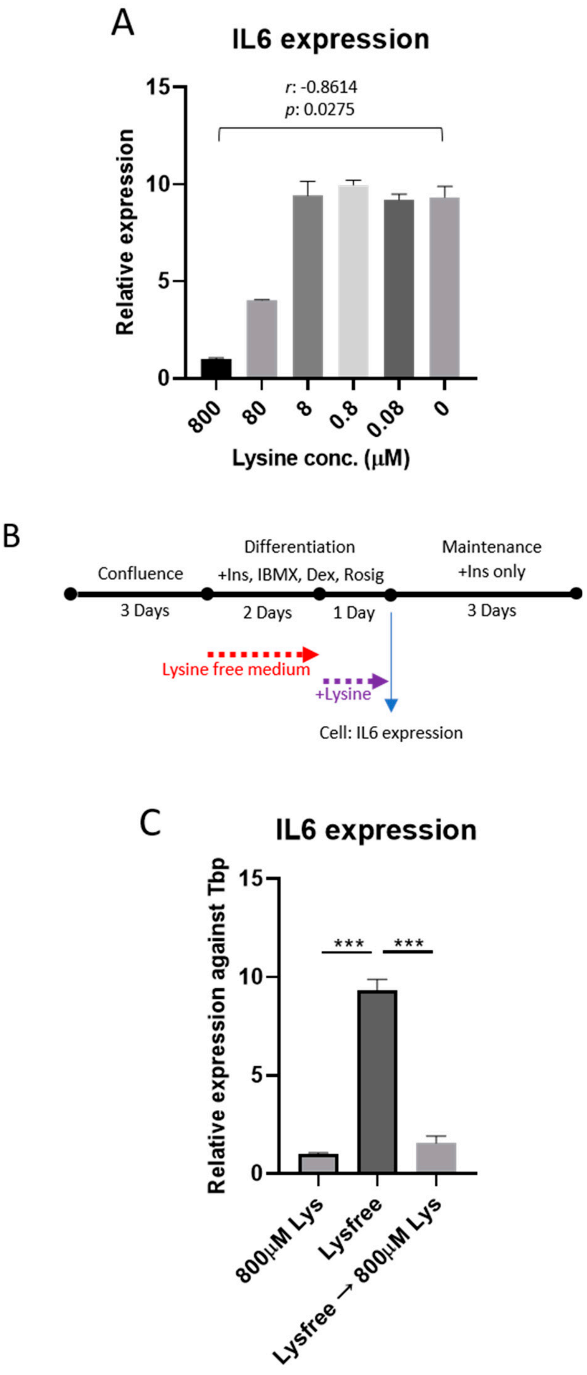

Supplementary Figure S6. The RNAseq data is matched with some published findings. (A) Lysine deprivation only downregulated CEBPa, but not CEBPb or CEBPd as reported by Kyoya et al [27]. (B-C) Trpv4, Trpm4, and Trpm5 were significantly downregulated (B) while Trpv1, Trpv2, and Trpc1 were significantly increased (C) in the differentiated adipocytes, as reported by Sun et al [28]. Data were expressed as means ± SE with n=3 in each condition. **p* < 0.05, ** *p* < 0.01 and ****p* < 0.001 using the One-way ANOVA followed by the Bonferroni test.

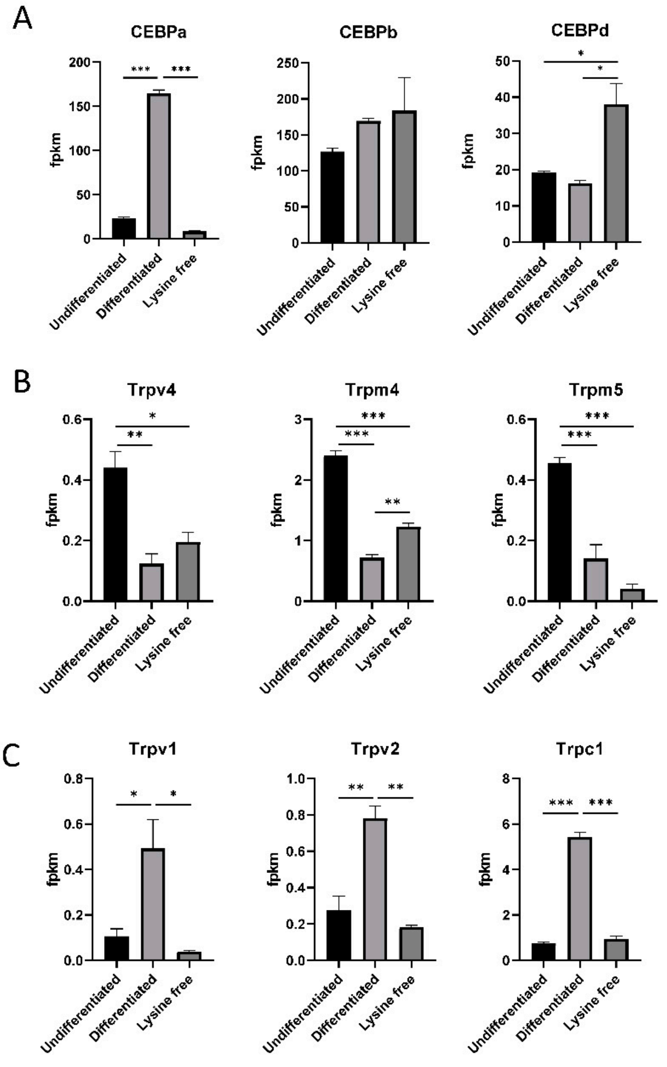

Supplementary Figure S7. Lysine deprivation suppressed lipolysis in 3T3-L1 cells. The three major lipases in the lipolysis pathway, adipose triglyceride lipase (ATGL), hormone-sensitive lipase (HSL), and monoacylglycerol lipase (MGL), which breaks triglyceride into glycerol and free fatty acid were presented. Data were expressed as means ± SE with n=3 in each condition. ***p < 0.001 using the One-way ANOVA followed by Bonferroni test.

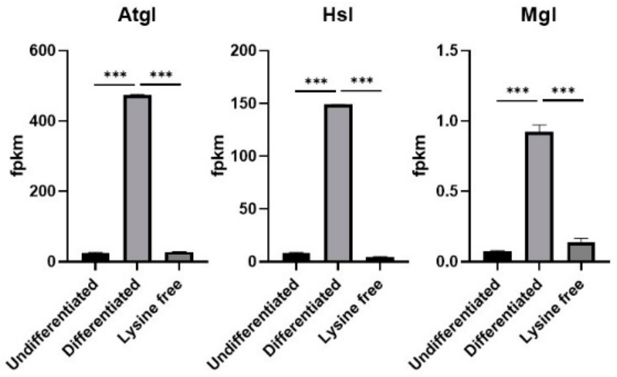

Supplementary Figure S8. Lysine deprivation mimicked transcriptomic change of IL6 supplementation. (A) Genes upregulated by IL6 treatment were found to have increased in cells under lysine-depletion. (B) Genes downregulated by IL6 were found decreased under lysine starvation. Data were expressed as means ± SE with n=3 in each condition. *$p < 0.05$, ** $p < 0.01$ and ***$p < 0.001$ using the One-way ANOVA followed by Bonferroni test.

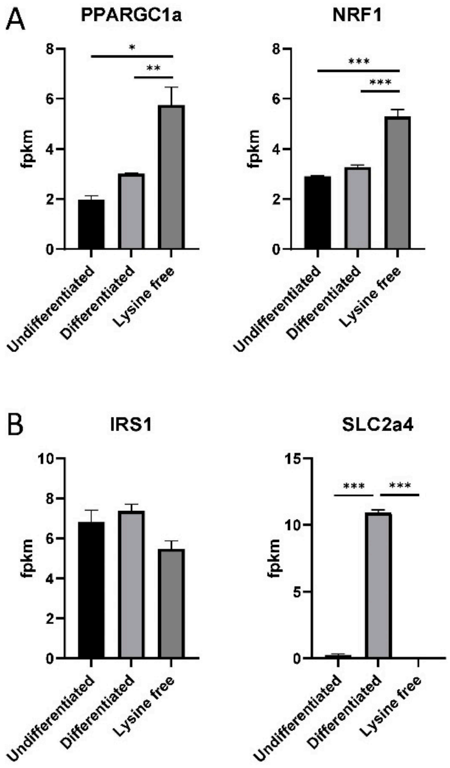

Supplementary Figure S9. Lysine deprivation activated lysosomal protease. (A) Lysosomal-associated membrane protein 1 (LAMP1) expression level. (B) Lysosomal proteinase legumain (LGMA), tripeptidyl peptidase 1(TPP1) and (C) the cathepsin (CTS) family expression level. Data were expressed as means ± SE with n=3 in each condition. *$p < 0.05$, ** $p < 0.01$ and ***$p < 0.001$ using the One-way ANOVA followed by Bonferroni test.

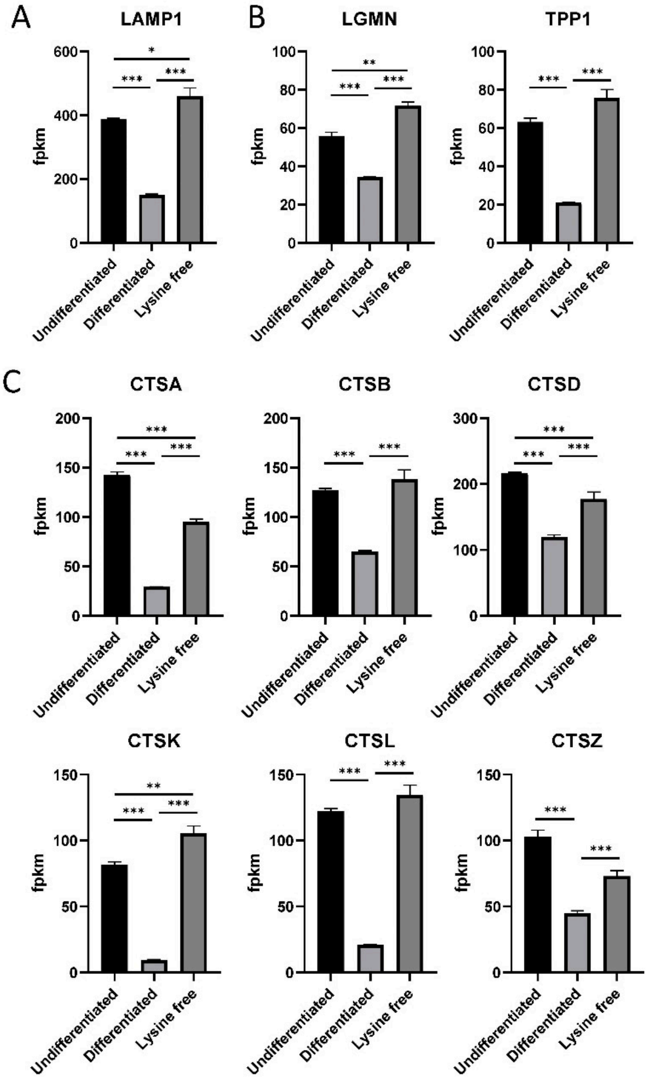

Supplement: Supplementary file 1 [file ijms-24-09402-s001.zip › ijms-2358954-supplementary.pdf]
